# Macrocirculatory Parameters and Oxygen Debt Indices in Pigs During Propofol Or Alfaxalone Anesthesia When Subjected to Experimental Stepwise Hemorrhage

**DOI:** 10.3389/fvets.2021.664112

**Published:** 2021-05-20

**Authors:** Andreas Lervik, Simen Forr Toverud, Jon Bohlin, Henning Andreas Haga

**Affiliations:** ^1^Department of Companion Animal Clinical Sciences, Faculty of Veterinary Medicine, Norwegian University of Life Sciences, Ås, Norway; ^2^Animal Health and Welfare Branch, Veterinary Inspectorate, Norwegian Armed Forces Joint Medical Services, Sessvollmoen, Norway; ^3^Division of Infection Control and Environmental Health, Department for Method Development and Analysis, Norwegian Institute of Public Health, Oslo, Norway; ^4^Center for Fertility and Health Analysis, Norwegian Institute of Public Health, Oslo, Norway

**Keywords:** pigs, anesthesia, TIVA, propofol, alfaxalone, dexmedetomidine, ketamine, hemorrhage

## Abstract

**Background:** Pigs are anesthetized when used for emergency procedures live tissue training (LTT) of civilian and military medical personnel or for experimental purposes, but there is a paucity in the literature regarding anesthesia of pigs for this purpose.

**Objective(s):** The main goals of the study were to compare oxygen debt, macrocirculatory parameters, and time to cardiac arrest between pigs in hemorrhagic shock and anesthetized with propofol-ketamine-dexmedetomidine or alfaxalone-ketamine-dexmedetomidine.

**Design:** A prospective, non-blinded randomized study design was used. Sixteen pigs were randomized in blocks of four to be anesthetized with either propofol-ketamine-dexmedetomidine (*n* = 8) or alfaxalone-ketamine-dexmedetomidine (*n* = 8) as a continuous infusion.

**Interventions:** Premedication with ketamine 15 mg kg^−1^ and midazolam 1 mg kg^−1^ was given i.m. Anesthesia was maintained with propofol 8 mg kg^−1^ h^−1^ or alfaxalone 5 mg kg^−1^ h^−1^ combined with ketamine 5 mg kg^−1^ h^−1^ and dexmedetomidine 4 μg kg^−1^ h^−1^ i.v. A stepwise, volume-controlled model for hemorrhage was created by exsanguination.

**Main Outcome Measures:** Indices of oxygen debt (lactate, base excess, and oxygen extraction), macrocirculatory (PR, SAP, DAP, MAP, and *CI*, SVI, and TPR) variables, and time to death was compared between groups.

**Results:** Pigs in the alfaxalone group had significantly higher SAP than pigs given propofol. No difference in other macrocirculatory variables or indices of oxygen debt could be found. A blood loss of 50% of the total blood volume or more was possible in most pigs with both anesthetic regimes.

**Conclusions:** Pigs anesthetized with propofol or alfaxalone combined with ketamine and dexmedetomidine tolerated substantial blood loss.

## Introduction

For emergency procedures live tissue training (LTT) of civilian and military medical personnel *Sus domesticus* is the most commonly used species (1). When anesthetizing pigs for LTT the animal must be unconscious and immobile, and proper antinociception should be provided. Hence the goals of general anesthesia should be fulfilled. To avoid premature death, the anesthetic regime should provide cardiovascular stability during surgery and tolerance to hemorrhage and hypovolemia. This will reduce the total number of animals used, and thereby adhering to the 3 R's principle. For training of human anesthesiologists, the model should ideally mimic a realistic emergency situation, enhancing the learning outcome. This could include using intravenous anesthetic agents to maintain anesthesia and preserving the cardiovascular response during episodes of hemorrhage. A conundrum when anesthetizing pigs for LTT is accomplishing all the above described aspects, as fulfilling one might influence the others.

In a previous study in pigs propofol, ketamine, and dexmedetomidine combined was found to maintain a stable cardiovascular status and provide antinociception (2). As a pilot investigation for the current experiment, 30% of the pigs' blood volume was removed but only a minimal chronotropic response was observed. In two other studies in pigs anesthetized with propofol and remifentanil exsanguination of 35–55% of the blood volumes increased the heart rate only by ~30% from baseline (3, 4). This is relatively modest compared with findings in pigs anesthetized with pure isoflurane anesthesia (5, 6).

Lack of chronotropic response to hypotension has also been shown after anesthetic induction with propofol in humans. In one study propofol depressed the baroreceptor reflex and sympathetic activity (7) and increases in vagal tone has also been a suggested cause (8). In fentanyl premedicated dogs, induction of anesthesia with alfaxalone decreased the heart rate significantly less than did propofol (9), while when the two drugs were compared for anesthetic maintenance no difference in cardiovascular variables such as, mean arterial blood pressure or heart rate could be found (10). We therefore wanted to compare the chronotropic response in pigs anesthetized by the previous studied anesthetic regime and when replacing propofol with alfaxalone.

Macrocirculatory parameters, including heart rate, arterial blood pressure and cardiac output (*CO*) are often used clinically to assess the severity of hypovolemia during hemorrhage, but are also deemed inadequate when used alone (11). Oxygen debt occurs when insufficient oxygen delivery results in a reduction of oxygen consumption, with subsequent cell death and organ system failure (12, 13). In experimental pigs subjected to hemorrhagic shock, oxygen debt is closely linked to the risk of death. The accumulation of metabolic acids calculated as base excess or specifically measured as lactate may be used to quantify oxygen debt (14).

The main goals of our study were to compare oxygen debt, macrocirculatory parameters, and time to cardiac arrest between pigs anesthetized with propofol-ketamine-dexmedetomidine or alfaxalone-ketamine-dexmedetomidine subjected to stepwise, volume-controlled model of hemorrhagic shock.

## Materials and Methods

### Ethics

Ethical approval was provided by the Norwegian National Animal Research Authority (FOTS ID 14277), Oslo, Norway (Advisor Marianne Waldum Furnes) on the 2nd of January 2018.

### Animals

The experiments were performed between the 22nd of January and 23rd of March 2018 at The Research Animal Facility of the Norwegian University of Life Sciences. Sixteen mixed breed pigs (Norwegian land race 50% and Duroc 50%), 10 castrated males and six females, were included. They originated from the Animal Production Experimental Centre of the University, with a median (range) age of 66 (52–73) days and a mean (SD) body weight of 24.9 (4.2) kg in the propofol group and 25.9 (3.7) kg in the alfaxalone group at the time of anesthesia. They were identified by using their existing ear tag numbers and housed with natural light-dark cycles and room temperature between 15 and 20°C and fed a commercial pig diet combined with free access to hay at the Research Animal Facility of the Norwegian University of Life Sciences for 14 days prior to the experiment. Health status was monitored at minimum once daily during the entire period. Prior to the current study, the pigs were used in another anesthesia study with a minimum wash out period of seven days in between.

### Study Design

A prospective, non-blinded, balanced, randomized study design was used. The 16 pigs were randomized in blocks of four to receive either propofol-ketamine-dexmedetomidine (*n* = 8) or alfaxalone-ketamine-dexmedetomidine (*n* = 8) by drawing paper notes.

### Anesthesia

Grain was withheld for approximately 12 h before premedication, and all pigs were deemed healthy based on a clinical examination before each experimental session.

Premedication with ketamine 15 mg kg^−1^ (Ketamine Le Vet 100 mg ml^−1^; Le Vet Beheer B.V., Holland) in combination with midazolam 1 mg kg^−1^ (Midazolam 5 mg ml^−1^; B. Braun, Germany) was given intramuscularly in the cervical muscles behind the ear. An intravenous catheter (Venflon Pro; Becton Dickinson Infusion Therapy, USA) was placed in an auricular vein. Anesthesia was induced with propofol (Propofol-Lipuro 20 mg ml^−1^; B. Braun, Germany) or alfaxalone (Alfaxan 10 mg ml^−1^; Jurox, Rutherford, Australia) to allow endotracheal intubation after topical application of lidocaine (Xylocaine 100 mg/ml spray, Aspen, Denmark) in the laryngeal area. They were placed in left lateral recumbency, covered with bubble wrap and external heat was provided with a forced air patient warming device (Bair Hugger, 3M, MN, USA) if the body temperature was <39.5°C. Volume controlled intermittent positive pressure ventilation was instituted with a rate of 20 breaths min^−1^ and tidal volume adjusted to maintain end tidal CO_2_ (*P*E′CO_2_) between 5.0 and 6.0 kPa before inducing hemorrhage. Ventilator settings was thereafter kept constant throughout the study.

Anesthesia was maintained with propofol 8 mg kg^−1^ h^−1^ or alfaxalone 5 mg kg^−1^ h^−1^, ketamine diluted to 50 mg ml^−1^ at 5 mg kg^−1^ h^−1^ and dexmedetomidine (Dexdomitor 0.5 mg ml^−1^, Orion Corporation, Finland) diluted to 50 μg ml^−1^ at 4 μg kg^−1^ h^−1^ i.v. Each drug was delivered by a separate syringe driver (Alaris GH Plus, BD Medical, Franklin Lakes, NJ, USA). The doses of propofol, ketamine and dexmedetomidine were based on the results from a previous study (2), whereas the alfaxalone dose was based on a pilot study in four pigs, where the infusion rate abolishing the motor response to an electrical nociceptive stimulus used in the previous study (2) was determined.

All pigs received a balanced electrolyte solution (Ringers acetate; Fresenius Kabi, Norway) i.v. at a rate of 0.33 ml kg^−1^ h^−1^ delivered by a volumetric infusion pump (Volumat Agilia; Fresenius Kabi, Norway). The total infused fluid volume including anesthetic drugs was 1.01 and 0.91 ml kg^−1^ h^−1^ in the alfaxalone and propofol group, respectively.

### Instrumentation, Monitoring, and Data Collection

A multiparameter anesthetic monitor was used (GE Carescape Monitor B650; GE Healthcare, Finland) to record heart rate, 3-lead electrocardiography, invasive systolic, mean and diastolic arterial blood pressure (SAP, MAP, DAP), pulse rate (PR) taken from the arterial blood pressure trace, arterial oxygen saturation, end-tidal CO2 (*P*E′CO_2_), inspired oxygen fraction, and esophageal temperature. Data were downloaded every 5 s using data collection software (iCollect Version 5.0, GE Healthcare, Finland).

A 10 cm, 6 Fr. introducer sheath (Percutaneous sheath introducer set; Arrow Int. Inc., USA) was placed in the external jugular vein under ultrasound guidance using a 13 MHz linear array transducer (SL 3323, Esaote, Italy) and preprogrammed settings for small parts in the ultrasound system (Esaote MyLab One, Esaote, Italy). A single lumen 60 cm, 6 Fr. balloon pulmonary artery catheter (Balloon Wedge Pressure Catheter; Arrow Int. Inc., USA) (PAC) attached to a pressure transducer (TruWave pressure monitoring transducer; Edwards Lifesciences Corp., USA) was inserted through the introducer sheath, and advanced into the pulmonary artery under observation of the characteristic waveforms and pressures. The pressure transducer was fixed at the level of the sternum and zeroed to atmospheric pressure.

A 20 cm thermistor tip, 5 Fr. thermodilution catheter (PiCCO Catheter; Pulsion Medical Systems SE, Germany) was placed by a modified Seldinger technique in the left tibial artery, and connected to a pressure transducer set (TruWave pressure monitoring transducer; Edwards Lifesciences Corp., USA) fixed at the level of the sternum and zeroed to atmospheric pressure, allowing continuous arterial blood pressure measurement, in addition to transpulmonary thermodilution measurement of *CO*.

To allow for rapid blood withdrawal, a 23 cm, 18-gauge catheter (Arterial Catheterization set, Arrow Int. Inc., USA) was placed percutaneously in the right femoral artery under ultrasound guidance using a modified Seldinger technique.

### Hemorrhage

**Figure 1:**
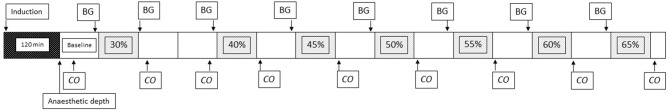
Timeline of the experiment. Figure showing timeline of the experiment. Evaluation of anesthetic depth, percentage of total blood volume removed, measurement of cardiac output (*CO*), and sampling time point for blood gases (BG) are shown. The first box (black) represents the 120 min from induction of anesthesia until start of the experiment. Each additional box represents 10 min.

Hemorrhagic shock was initiated ~120 min after induction of anesthesia. The total blood volume was estimated as 65 ml/kg body weight (15). The blood volume to be withdrawn was taken from the femoral arterial catheter using a 60 ml syringe, a closed collection system, a 3-way stop-cock and a stopwatch to achieve a steady exsanguination rate. Thirty percent (H30) of the total blood volume was first removed over 10 min, followed by a 20 min period to allow for compensation. Thereafter 10% of the total blood volume (H40) was withdrawn over 10 min, followed by a 10 min period to allow for compensation. Thereafter 5% of the total blood volume was withdrawn every 10 min (H45, H50, and so on), followed by a 10 min period to allow for compensation between each period until cardiac arrest occurred (Figure 1). Cardiac arrest was defined as the first time point when *P*E′CO_2_ was below 1.5 kPa with a concurrent pulse pressure below 10 mmHg.

### Cardiovascular Evaluation

*CO* was measured prior to and after H30 and H40. Subsequent measurements of *CO* were then performed only after each period of hemorrhage (Figure 1).

For the transpulmonary thermodilution measurement, 10 ml of cold saline was injected as a rapid bolus through an injectate temperature sensor housing (Pulsion Medical System SE, Germany) into the side port of the jugular venous introducer sheath. The *CO* measurement was repeated three times at each time point. If a measurement deviated more than 10% from the mean of these measurements, additional measurements were performed until there were three measurements within this range. To standardize injected fluid volume between groups all injections were performed even if a *CO* could not be measured at the low flow states during shock.

### Evaluation of Anesthetic Depth

Anesthetic depth was evaluated after the baseline measurement of *CO*, prior to induction of hemorrhage (Figure 1).

Clinical signs of anesthetic depth were evaluated and scored using a binary scoring system by the same investigator, eye position (ventral = 0, central = 1), nystagmus (present = 0, absent = 1), palpebral reflex (present = 0, absent = 1), and corneal reflex (present = 0, absent = 1) were assessed. Thereafter mechanical nociceptive stimulation was applied to the lateral dewclaw of the right front limb by a latex-coated forceps with a clamping area 1 × 1 cm. The applied pressure was monitored with a spring balance attached to one forceps arm at a point equal distance from, and on the opposite side of the articulation as the clamping jaws. The force applied at clamping was 100 N and maximum clamping time for each stimulus was 59 s. Stimulation was stopped at withdrawal of the limb or if vigorous movement in other limbs or whole-body movement was observed. The withdrawal was scored as present (=0) or absent (=1). A summarized anesthetic depth score created for this study ranging from 0 to 5 was then calculated.

In seven pigs a two-channel referential electroencephalogram (EEG) was recorded using needle electrodes (Aiglette, Technomed Europe, Netherlands) placed 1 cm caudal to the lateral angle of the eye and 1 cm medial to the temporal line bilaterally. These two electrodes were referred to an electrode placed in the median plane 2 cm caudal to the recording electrodes. A ground electrode was placed caudal to the atlas wing. The resistance between the recording electrodes was kept below 3 kΩ. The electrodes were connected to an EEG monitor (A-1.000^TM^, Aspect Medical Systems, USA). The monitor filters were set as follows - high frequency filter: 50 Hz, 50/60 Hz filter: 50 Hz and low frequency filter: 2.0 Hz. The monitor automatically detected burst suppression and calculated suppression ratio (BSR) as the percentage of epochs in the previous 63 s where the EEG signal was considered suppressed as a running average every 5 s.

### Blood Sampling and Analysis

One ml of arterial and mixed venous blood was simultaneously sampled from the arterial and PAC before hemorrhage (baseline). Thereafter blood was withdrawn after H30 and then before each period of hemorrhage (Figure 1). Samples were drawn into heparinized blood gas syringes (Pico 70; Radiometer, Denmark), and analyzed within 30 min using a bench top blood gas analyzer (ABL 800 Flex; Radiometer, Denmark).

### Data Analysis and Statistics

A power analysis was performed prior to the study. A difference in arterial lactate of at least 2.1 mmol/L after H50 was considered as clinically significant since this difference previously has been associated with an increase in mortality from 25 to 50% (16). In the same study a standard deviation of 1.8 mmol/L was found. Aiming at a beta of 0.8 and an alpha of 0.05, a total number of 26 pigs was needed. Based on this we decided to include a total of 32 pigs, but also to perform an interim statistical analysis including a new sample size calculation based on the observed difference in lactate after the first 16 pigs had been enrolled.

A database was created in Microsoft excel and additional calculations were made (Microsoft Corp., NM, USA):

Haemoglobin concentration (Hb) = (Hb_arterial_ + Hb_venous_)/2
Body surface area (BSA) m^2^ = 0.0734 × BW^0.656^
Cardiac index (*CI*) = *CO*/BSA
Stroke volume index (SVI) = (*CO*/HR)/BSA
Total peripheral resistance (TPR) = 80 × MAP/*CO*
Content of oxygen in arterial blood (CaO_2_) = (1.34 × Hb × SaO_2_) + (0.025 × PaO_2_)
Content of oxygen in mixed venous blood (CvO_2_) = (1.34 × Hb × SvO_2_) + (0.025 × PvO_2_)
Oxygen extraction (OE) = (CaO_2_-CvO_2_)

Graphical and further statistical analysis was performed using statistical software (JMP Pro 15.0.0, SAS, NC, USA, and R with the “nlme” package).

For comparison of indices of oxygen debt, including arterial lactate concentration (lactate), arterial base excess (BE) and OE, and macrocirculatory parameters, including PR, SAP, DAP, MAP, *CI*, SVI, and TPR, linear mixed effect model “lme” with restricted maximum likelihood (REML) was used. All models were fitted with pig with respect to time as a random slope effect, while several covariates were tested against both a null model having only the random slope effect and no covariates and a null model having neither covariates nor random effects. The latter model was fitted using the “gls” function using REML. The Akaike Information Criterion (AIC) was used to examine goodness of fit of all models. Model fit to data and normality of model residuals were examined using distributional plots of the residuals resulting from the models. All model testing statistics, without exception, favored the inclusion of random slope effects.

Time to cardiac arrest was compared between groups using a Kaplan Meier analysis. α was set to 0.05.

## Results

Data are given as mean ± SD unless otherwise stated.

Based on the interim analysis after the inclusion of eight pigs in each group, with a difference in lactate of 0.94 ± 3.58 mmol/L at H50, a total number of 458 pigs were needed to find a significant difference in lactate at H50. No further pigs were thus included.

The median (range) summarized anesthetic depth score was 4 (3–5) in both groups, while the median (range) BSR was 3 (0–27) in the propofol group, and 2 (1–53) in the alfaxalone group. EEG was evaluated in the last seven pigs of the experiment. One of eight and three of eight pigs had a positive withdrawal reflex in response to clamping of the dewclaw in the propofol and alfaxalone group, respectively. The one pig in the propofol group displayed a BSR of 3, while the two pigs with a positive withdrawal reflex in the alfaxalone group had a BSR of 1 and 2. In the third pig EEG was not evaluated.

**Figure 2:**
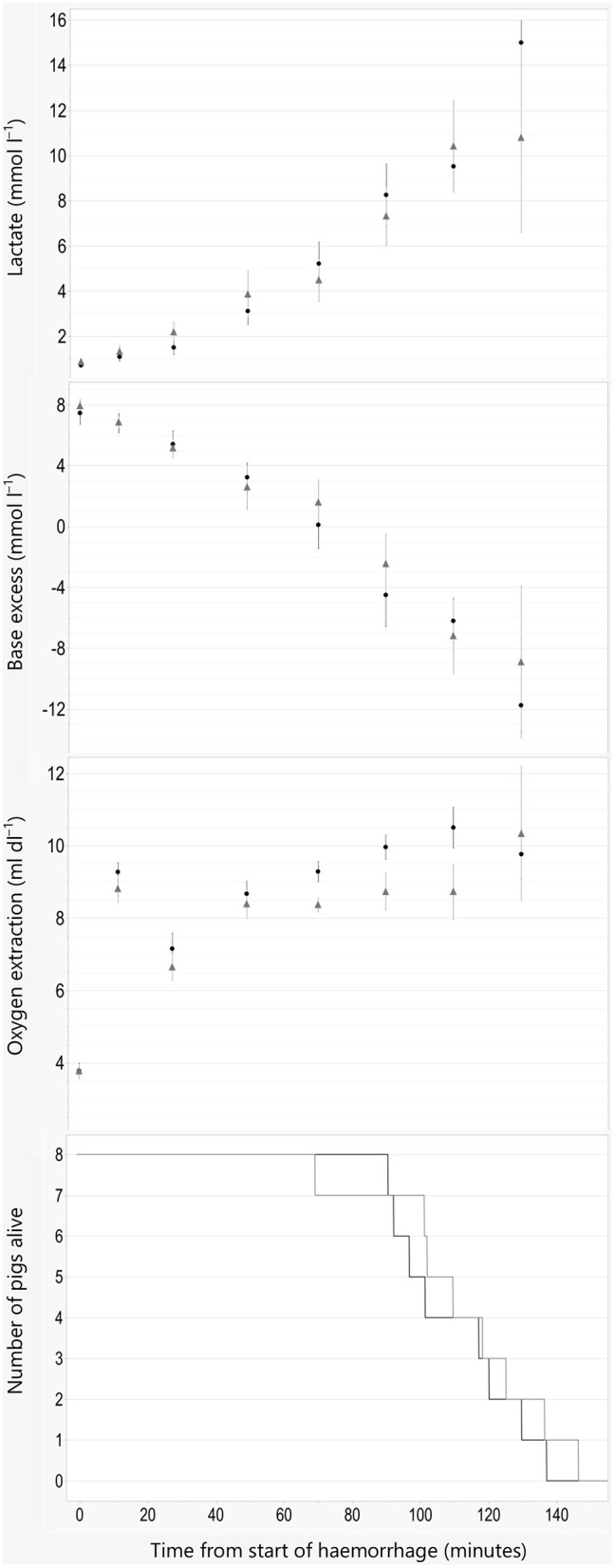
Indices of oxygen debt and time of death. Data are plotted against time in minutes from start of hemorrhage. Arterial blood lactate concentration, base excess, and oxygen extraction are illustrated as Mean ±SEM, and numbers of pigs alive are illustrated as lines. Black line and filled circles represent pigs anesthetized with propofol 8 mg kg^−1^ h^−1^. Gray line and triangles represent pigs anesthetized with alfaxalone 5 mg kg^−1^ h^−1^. In addition, all pigs were administered ketamine 5 mg kg^−1^ h^−1^ and dexmedetomidine 4 μg kg^−1^ h^−1^ IV.

Time until death is shown in Figure 2. No significant difference in time to death was found between groups (*P* = 0.56). Withdrawn median (range) blood volume at the time of cardiac arrest was 56.8 (50.5–64.5) and 57.3 (45–65)% of the total blood volume in the propofol and alfaxalone group, respectively.

There was a statistically significant association between MAP and treatment when adjusting for weight (8.54, 95% CI 0.88–16.3, *P* = 0.04, AIC = 6,178), where alfaxalone was associated with higher MAP. Adjusting for sex reduced the effect and AIC slightly (7.32, 95% CI −0.42–15.06, *P* = 0.08, AIC = 6,174), suggesting that the effect of treatment on MAP was weak.

An effect was detected for SAP including weight (13.62, 95% CI 3.72–23.52, *P* = 0.02, AIC = 6,562) and both weight and sex (11.76, 95% CI 2.2–21.32, *P* = 0.03, AIC = 6,557), where treatment with alfaxalone was associated with higher SAP.

For DAP the effect was at best weak when adjusting for weight (6.68, 95% CI −0.1–13.46, *P* = 0.07, AIC = 5,856) and non-significant when adjusting for both weight and sex (5.71, 95% CI −1.25–12.67, *P* = 0.13, AIC = 5,852).

For all other models, including lactate, BE, OE, PR, *CI*, SVI, and TPR the effect of treatment was not significant (*P* > 0.05), regardless of covariates included, and therefor omitted.

**Figure 3:**
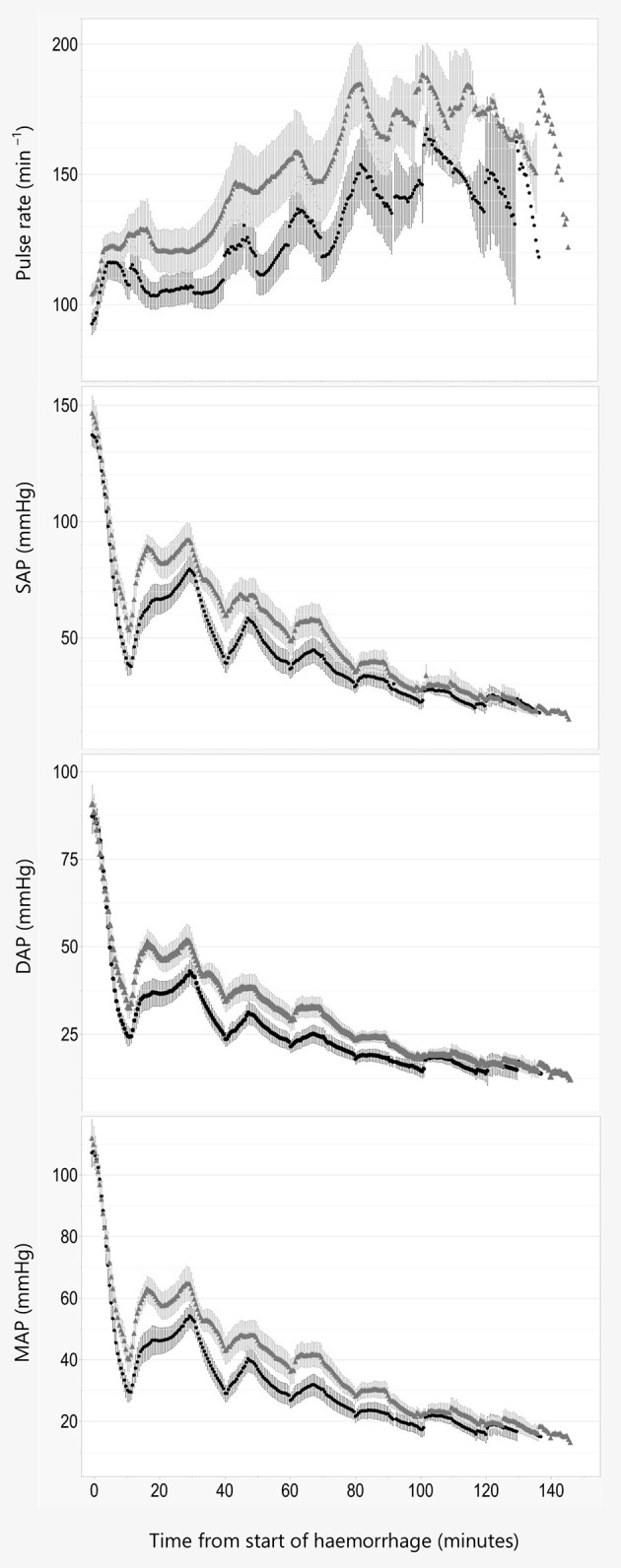
Pulse rate and blood pressure. Data are plotted against time in minutes from start of hemorrhage. Pulse rate, systolic (SAP), diastolic (DAP), and mean (MAP) arterial blood pressure are illustrated as Mean ± SEM, and black line and filled circles represent pigs anesthetized with propofol 8 mg kg^−1^ h^−1^. Gray line and triangles represent pigs anesthetized with alfaxalone 5 mg kg^−1^ h^−1^. In addition, all pigs were administered ketamine 5 mg kg^−1^ h^−1^ and dexmedetomidine 4 μg kg^−1^ h^−1^ IV.

**Figure 4:**
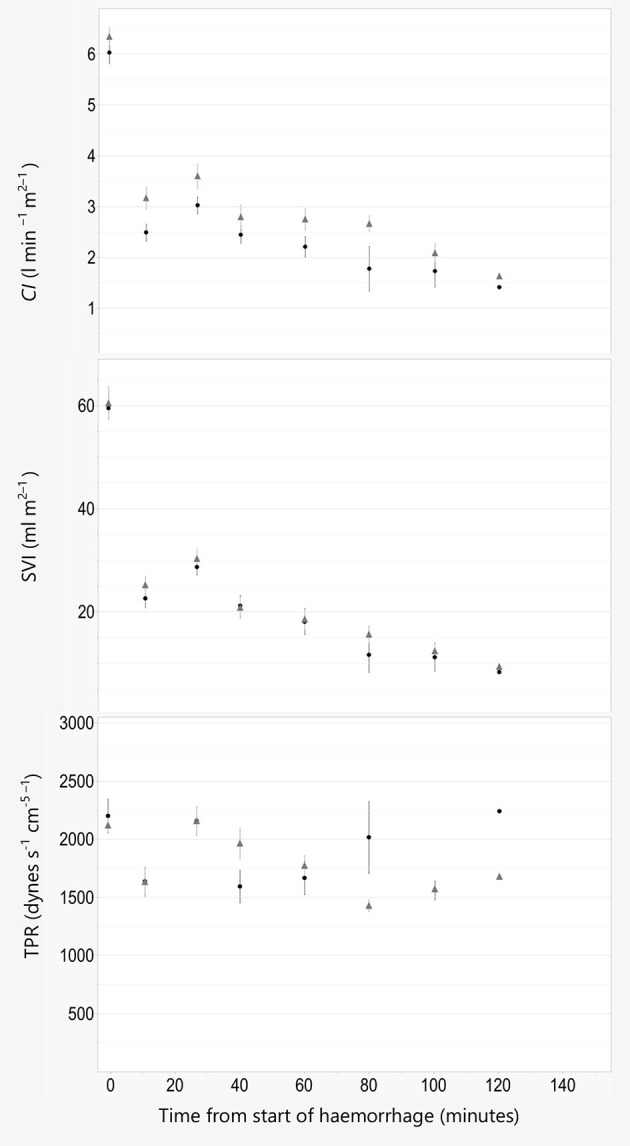
Cardiac index, stroke volume index, and total peripheral resistance. Data are plotted against time in minutes from start of hemorrhage. Cardiac index (*CI*), stroke volume index (SVI), and total peripheral resistance (TPR) are illustrated as Mean ± SEM, and black line and filled circles represent pigs anesthetized with propofol 8 mg kg^−1^ h^−1^. Gray line and triangles represent pigs anesthetized with alfaxalone 5 mg kg^−1^ h^−1^. In addition, all pigs were administered ketamine 5 mg kg^−1^ h^−1^ and dexmedetomidine 4 μg kg^−1^ h^−1^ IV.

Graphical description of oxygen debt indices and macrocirculatory parameters are shown in Figures 2–4.

## Discussion

Pigs anesthetized with either propofol-ketamine-dexmedetomidine or alfaxalone-ketamine-dexmedetomidine displayed a similar resilience to blood loss. A non-lethal volume loss of 50% or more of the total blood volume was possible in most pigs, even without resuscitation efforts. This volume of blood loss exceeds the volume loss defining class IV hemorrhage in the Advanced trauma life support classification of hypovolemic shock (17); a level of blood loss that is associated with severe physiological derangements and mortality in humans, often reproduced in the laboratory setting (18, 19) and encountered during LTT.

Oxygen debt is strongly associated with cellular damage, apoptosis and mortality in animal models of hemorrhagic shock (14). Both increased lactate and decreased BE correspond well with increased oxygen debt. The LD50 for oxygen debt in pigs subjected to hemorrhage occur at a BE reduction of −15.3 mmol/L and a lactate concentration increase of 7.7 mmol/L (16). This corresponds to values of base excess and plasma lactate where 50% mortality occurred after ~55% blood loss in the current study. A difference between the two groups was found in blood pressure, but not for base excess or blood lactate. The changes found for indices of oxygen debt are similar to the results in the study by Rixen et al. (16), where anesthesia was maintained with etomidate and fentanyl.

Pigs anesthetized with alfaxalone had a significantly higher systolic blood pressure than pigs given propofol. This could result from higher *CO* due to a higher PR og SVI, or a higher TPR in the alfaxalone group. Concurrently no statistical difference could be established for the other macrocirculatory parameters examined, including *CO* and TPR. Upon visual inspection of the trend curves for these parameters we do however consider the difference in systolic pressure to be a consequence of a higher PR and thus *CO* in the alfaxalone treated pigs, rather than a change in SVI or TPR, as these are more similar between the two groups.

Our observation from the previous pilot study could not be reproduced, as pigs in both groups displayed increasing pulse rate with increasing hemorrhage volume. However, the variation in pulse rate was large in both groups, and pigs anesthetized with alfaxalone seem to maintain a stronger chronotropic response to hypovolemia than pigs given propofol. This is similar to findings in a previous investigation comparing alphaxalone/alphadolone and propofol when used to maintain anesthesia in pigs subjected to hemorrhage, where a higher heart rate was found at baseline after 2 h of drug infusion, and after a blood loss of 40 ml kg^−1^ (20).

Ketamine and dexmedetomidine are drugs known to influence cardiovascular function. Ketamine has been shown to increase heart rate and thereby blood pressure (21), while dexmedetomidine is known to induce bradycardia in several species, including pigs (22–24). In a previous publication in normovolemic pigs dexmedetomidine did not produce the expected decrease in heart rate (2), a finding that was later reproduced with pigs in isoflurane anesthesia (25). The reason for this surprising finding is not known. In the same publication we found a slight increase in TPR when dexmedetomidine was infused, while TPR remained relatively unchanged in the current experiment despite significant blood loss. The cause of this vascular unresponsiveness cannot be elucidated in our study. The cardiac index found at baseline in this experiment was similar in both groups, and also similar to that found in our previous publication in normovolemic pig given either fentanyl or dexmedetomidine in combination with propofol and ketamine (2). This leads us to believe that the cardiovascular effects of ketamine and dexmedetomidine are similar in pigs given either propofol or alfaxalone. At the same time a cardiovascular comparison of the two drugs could very well have been different without the addition of ketamine and dexmedetomidine.

A fixed volume, stepwise hemorrhage model was used in our study. The advantage of fixed volume hemorrhage is that physiological responses and compensation can be compared between groups (15). At the same time the effect of hemorrhage on cardiovascular performance and oxygen debt is not controlled and may vary between individuals. Also, clinical hemorrhage is not linear, and a previous study found that the speed of hemorrhage can influence the physiological response observed (18). A weakness of that study was however the large variation in the isoflurane concentrations used. Despite this, it seems important to interpret results from studies of experimental hemorrhage in the light of the hemorrhage model used. The stepwise model with a decreasing loss of volume over time and time for compensation was used to resemble clinical hemorrhage during for example live tissue training more closely than a continuous hemorrhage model would do.

Defining equipotent doses of anesthetic agents and similar anesthetic depth is challenging. To compare anesthetic depth a simple scoring system was used based on common clinical indicators of anesthetic depth in pigs, in conjunction with EEG in seven of the animals. EEG was added to the study protocol after the inclusion of nine animals as one of the pigs displayed a positive response to mechanical nociceptive stimulation, in addition to a positive corneal reflex being observed in some pigs. EEG indicated that the cerebrocortical depression was profound with both anesthetic regimes, with several pigs displaying burst suppression (26). The use of ketamine in our anesthetic protocol could have influenced the EEG results, as ketamine traditionally has been known to increase EEG activity (27). The combined effects of ketamine and GABA-agonists or inhalational anesthetics on the EEG do however seem to be more complex, as the addition of ketamine also has been shown to induce burst suppression in rats (28). In addition, the anesthetics depth score was similar in both groups before induction of hemorrhage. With progressive hemorrhage plasma concentrations of both propofol and alfaxalone increased in our study, with an increasing variation in plasma concentrations of both propofol and alfaxalone (unpublished observations).

*CO* measurement using transpulmonary thermodilution has shown good accuracy when compared to techniques using thermistor catheters in the pulmonary artery, and a comparable precision has also been shown in pigs when comparing the two methods (29–31). At the same time variations in agreement was documented in a study comparing the two methods at different levels of *CO* (32). All measurements in our study were performed in triplicates, and the need for repeated measurements due to variations in the three measurements was very low. We did however encounter difficulties obtaining measurements at very low flow states, resulting in missing *CO* data toward the end of each experimental session. This was not unexpected and is probably due to recirculation and loss of indicator, leading to overestimations of *CO* followed by failing measurements (33–35). If a false increase in *CO* occurred at lower flow states, this could make it harder to detect differences between the anesthetic agents examined.

In this study lactate was considered the primary outcome variable and was used for interim sample size calculation. If more animals had been included a statistical difference may have been found for macrocirculatory variables. However, based on our results, a possible difference would likely have been small. The rationale for an interim analysis was a to minimize the total number of animals being sacrificed, which may have resulted in not detecting subtle statistical differences in other variables.

The reported blood volume in pigs shows some variation in different publications (15, 36, 37), and the reported hemorrhage volume tolerated in our pigs is based on a total blood volume of 65 ml kg^−1^. Also, the pigs in our study were not splenectomized. Splenic contraction can increase the circulating volume and oxygen carrying capacity in pigs during hemorrhage, and splenectomy may influence the physiological response to blood loss (38). A difference in splenic volume has also been reported with different anesthetic drugs in dogs (39), but to our knowledge the effect of propofol or alfaxalone on splenic size in pigs has not been examined. For the comparison between groups in our study this likely has no consequence. All pigs used were from two litters and were block randomized to reduce the interindividual variation between groups.

An additional limitation of our study could be the transferability into a clinical situation in pigs anesthetized for LTT or experimental surgery. Resuscitation efforts where not performed in our pigs. Hence, the cardiovascular response to interventions such as intravenous fluid therapy or vasoactive drugs remains unknown, and potential differences between treatments remains undiscovered. In addition, only a small volume of fluids where administered not to change the volume status of the pigs during the experiment. One could however argue that the infused fluid volume was to low, and thus not mimicking a realistic clinical situation. Another limitation is that the nociceptive and cardiovascular response to surgical trauma was not examined. The anesthetic regime has been used by the authors in pigs, and we consider the cardiovascular stability to be excellent during invasive experimental surgical procedures (personal observations).

In conclusion, total intravenous anesthesia with either propofol-ketamine-dexmedetomidine or alfaxalone-ketamine-dexmedetomidine allows for substantial blood loss in pigs used in experimental settings. Pigs anesthetized with alfaxalone seem to maintain a higher systolic blood pressure than pigs given propofol, but we could not find differences in other macrocirculatory variables or indices of oxygen debt.

## Data Availability Statement

The raw data supporting the conclusions of this article will be made available by the authors, without undue reservation.

## Ethics Statement

Ethical approval was provided by the Norwegian National Animal Research Authority (FOTS ID 14277), Oslo, Norway (Advisor Marianne Waldum Furnes) on the 2nd of January 2018.

## Author Contributions

AL and HH contributed to conception and study design. AL, HH, and SF prepared and performed the experiments including data collection. AL organized the data base. AL, HH, and JB performed the statistical analyses. AL and JB drafted the manuscript. All authors participated in preparing the manuscript, read, and approved the final manuscript.

## Conflict of Interest

Dechra Veterinary Products provided the alfaxalone used in this study. The authors declare that the research was conducted in the absence of any commercial or financial relationships that could be construed as a potential conflict of interest.
